# Safety and feasibility of optimized transcranial direct current stimulation in patients with mild cognitive impairment due to Alzheimer’s disease: a multicenter study protocol for a randomized controlled trial

**DOI:** 10.3389/fneur.2024.1356073

**Published:** 2024-04-10

**Authors:** TaeYeong Kim, Dong Woo Kang, Jhosedyn Carolaym Salazar Fajardo, Hanna Jang, Yoo Hyun Um, Sunghwan Kim, Sheng-Min Wang, Donghyeon Kim, Hyun Kook Lim

**Affiliations:** ^1^Research Institute, Neurophet Inc., Seoul, Republic of Korea; ^2^Department of Psychiatry, Seoul St. Mary’s Hospital, College of Medicine, The Catholic University of Korea, Seoul, Republic of Korea; ^3^Department of Psychiatry, St. Vincent’s Hospital, College of Medicine, The Catholic University of Korea, Seoul, Republic of Korea; ^4^Department of Psychiatry, Yeouido St. Mary’s Hospital, College of Medicine, The Catholic University of Korea, Seoul, Republic of Korea

**Keywords:** brain modeling, cognitive function, mild cognitive impairment, non-invasive brain stimulation, transcranial direct current stimulation

## Abstract

**Introduction:**

Transcranial direct current stimulation (tDCS) may effectively preserve and improve cognitive function in patients with mild cognitive impairment (MCI). Research has shown that Individual brain characteristics can influence the effects of tDCS. Computer three-dimensional brain modeling based on magnetic resonance imaging (MRI) has been suggested as an alternative for determining the most accurate tDCS electrode position based on the patients’ individual brain characteristics to enhance tDCS effects. Therefore, this study aims to determine the feasibility and safety of applying tDCS treatment using optimized and personalized tDCS electrode positions in patients with Alzheimer’s disease (AD)-induced MCI using computer modeling and compare the results with those of a sham group to improve cognitive function.

**Method:**

A prospective active-sham group feasibility study was set to recruit 40 participants, who will be randomized into Optimized-tDCS and Sham-tDCS groups. The parameters for tDCS will be 2 mA (disk electrodes *R* = 1.5 cm) for 30 min during two sets of 15 sessions (2 weeks of resting period in between), using two electrodes in pairs. Using computer modeling, the tDCS electrode positions of each participant will be personalized. Outcome measurements are going to be obtained at three points: baseline, first post-test, and second post-test. The AD assessment scale-cognitive subscale (ADAS-Cog) and the Korean version of Mini-Mental State Examination (K-MMSE), together with other secondary outcomes and safety tests will be used.

**Discussion:**

For the present study, we hypothesize that compared to a sham group, the optimized personalized tDCS application would be effective in improving the cognitive function of patients with AD-induced MCI and the participants would tolerate the tDCS intervention without any significant adverse effects.

**Clinical trial registration**: https://cris.nih.go.kr, identifier [KCT0008918].

## 1 Introduction

Alzheimer’s disease (AD) is the most common form of dementia in the elderly (1). It is characterized by progressive memory loss, along with other cognitive impairments (CI) and executive function loss (2). Mild cognitive impairment (MCI) is considered the preclinical stage of AD, with objective CI, functional independence, and a 5–10 percent annual progression rate to dementia (2, 3). Due to the deposition levels of Amyloid-β (Aβ), which have become an indicator to determine the likelihood of developing AD, some individuals have MCI induced by AD (4). Research has found that Aβ might accumulate even before any MCI symptoms appear, meaning that the probability of developing AD existed even before the MCI was diagnosed (4). This means that the neurodegenerative process is already underway, and to date, there is little evidence on the effectiveness of treatments for Alzheimer’s disease and interventions aimed at controlling the disease.

Diverse pharmacological and physical therapies have been studied for MCI; unfortunately, to date, no consistent reports of long-term effectiveness have been published (5). However, increasing evidence suggests that transcranial direct current stimulation (tDCS) may be an effective add-on therapy to improve cognitive function in patients with MCI (2, 6, 7). tDCS is a non-invasive brain stimulation technique that involves placing electrodes on the scalp to apply a weak direct current to the brain cortex with the intent of modulating brain excitability (2). It influences the cognitive and motor functions associated with the stimulated cortical region by modulating the resting membrane potential (8). During the administration of tDCS, depolarization or hyperpolarization of the neuronal membrane of target neurons may be induced according to the polarity of the electrode (anode or cathode), intensity, and time of application (2, 9, 10).

Research has shown that tDCS improves the cognitive state of patients with MCI when applied at an intensity of 2 mA for either 20- or 30 min over periods of 10–20 days (5, 11, 12), showing the potential of tDCS to be used during long-term interventions. According to previous research, tDCS has also demonstrated positive outcomes during long-term interventions for patients with disorders such as depression. In a study involving individuals with major depressive disorder, 15 sessions of anodal tDCS were initially administered, with participants having the option to continue for an additional 15 sessions. The results indicated that those who extended the intervention experienced more lasting benefits than those who completed the intervention after the initial 15 sessions (13). While the past study focused on a different population, it highlights the significance of prolonged tDCS interventions. Hence, assessing the safety and feasibility of tDCS for at least 30 days in patients with MCI is necessary for broader future applications.

Moreover, the effects of tDCS depend not only on the stimulation parameters, but also on distinct individual brain characteristics. A study that included participants with MCI and major neurocognitive disorders applied tDCS at 2 mA for 20 min twice a day for 5 consecutive days but found no significant improvement in cognitive function as assessed through the Mini-Mental State Examination (MMSE) and Alzheimer’s dementia assessment scale–cognitive subscale (ADAS-Cog). According to the authors, the results may have been influenced by various factors, such as a small sample size and the target population, including participants in different stages of neurocognitive impairment (14).

This observation is plausible, given that the progression of MCI disorders affects white matter structural integrity (15). A study found, by using magnetic resonance imaging (MRI), that brain areas with greater preserved white matter structural integrity may be associated with better tDCS outcomes in healthy adults and individuals with CI (16, 17). Furthermore, the structure of the gyri and sulci, amount and distribution of cerebrospinal fluid (CSF), and thickness of the scalp and skull influence the electrical field (E-field) exerted by tDCS on cortical neurons and their subsequent excitability (16, 18). A systematic review published in 2023 recommended that tDCS research in patients with CI should consider individual brain characteristics by developing individually optimized tDCS procedures (19). Also, two previous studies investigating the effects of a combination of tDCS and cognitive training in patients with MCI provided evidence that tDCS affects cognitive changes in the patients, but did not find statistically significant differences with sham tDCS due to a small sample size and did not prove elevated tDCS effect on cognitive training benefits (20, 21). Thus, to determine the feasibility of tDCS as an add-on therapy for MCI induced by AD, brain models based on patients’ individual brain characteristics should be employed for focalized treatment, guided by the resulting E-field outcomes of the brain models.

Brain models require the analysis of individual three-dimensional (3D) T1 MRI to recreate realistic personalized 3D head models. These models are reconstructed from structural MRI and include the main brain tissues (skin, skull, cerebral gray matter and white matter, cerebellum gray and white matter, CSF, and ventricles), specific conductivity assumptions, and electrode properties (22). They enable researchers to determine the E-field using tDCS based on individual brain characteristics, thereby facilitating optimized tDCS applications (23). However, the creation of these models is time consuming and requires the use of multiple software tools to construct an accurate model, which limits their practical use in common clinical settings (23).

Therefore, an accessible software capable of performing brain structure segmentation based on MRI to calculate the E-field induced by tDCS was developed to facilitate the maximal stimulation effect based on individual unique brain characteristics (24). This software has been used previously in research and is expected to facilitate the use of optimized tDCS in clinical settings in the near future (25).

Considering all the above-mentioned studies, the present feasibility randomized double-blind controlled trial study protocol aims to outline the study methods and resources required to determine the feasibility and safety of a treatment for patients with MCI induced by AD with personalized optimized tDCS for the future design of a full-powered trial. The specific aims are to (1) determine the feasibility of optimized tDCS in the treatment of patients with MCI induced by AD for the improvement of cognitive function compared to a sham group, (2) determine the safety of using optimized tDCS in patients with MCI induced by AD, and (3) obtain valid data to sustain the calculation of a sample size for a fully powered trial, should tendencies of efficacy be present.

## 2 Methods and analysis

### 2.1 Trial design

**Figure 1:**
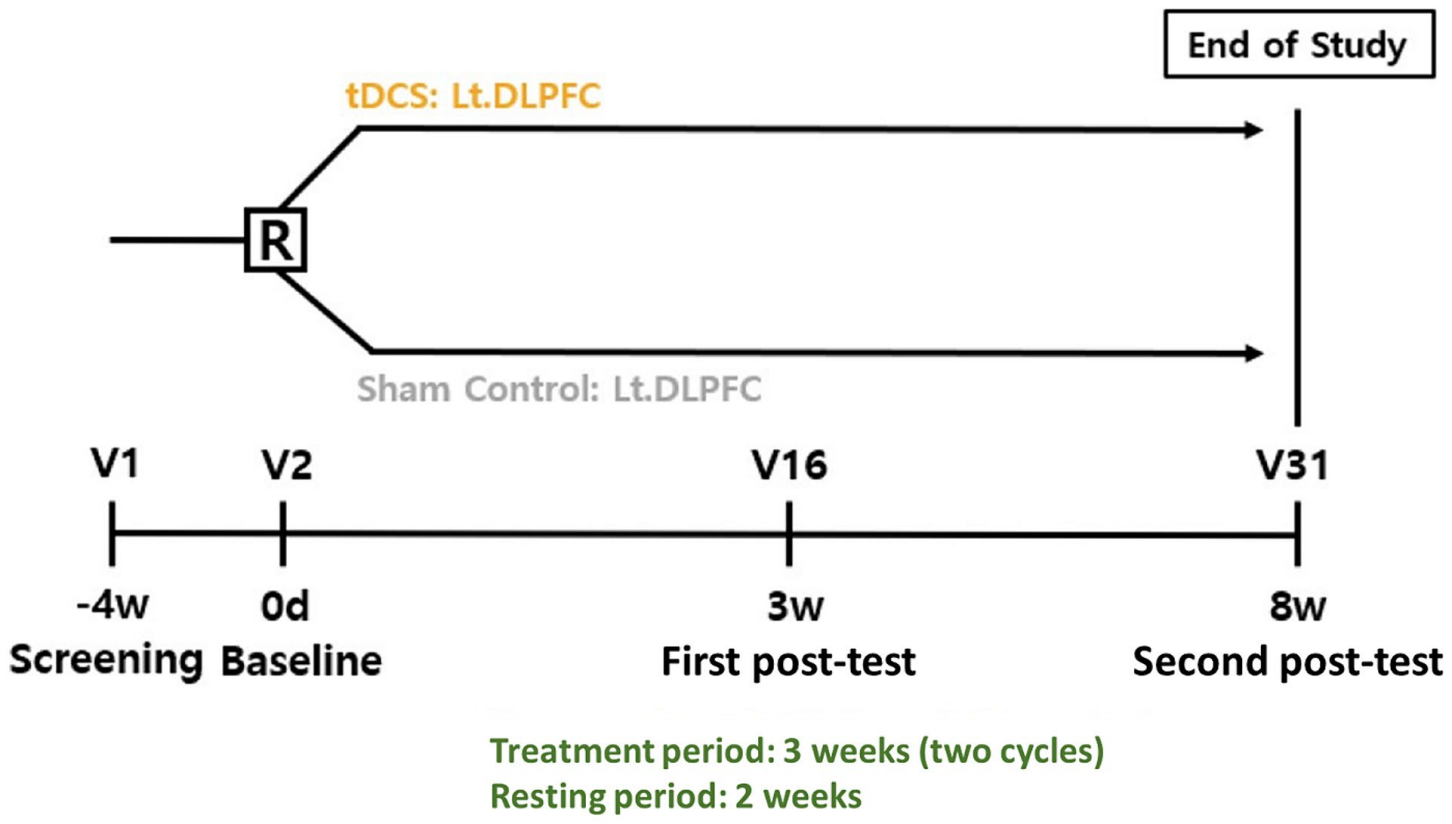
Study design R, randomization; tDCS, transcranial direct current stimulation; Lt DLPFC, left dorsolateral prefrontal cortex; V, visit.

The trial design is a prospective, multicenter, double-blind, randomized, active/sham group feasibility study. The active control group named the optimized-tDCS group will be compared with the sham-tDCS group to determine the feasibility and safety of optimized tDCS in the treatment of patients with MCI induced by AD. The intervention will be divided into two sets of 15 sessions (five times per week for 3 weeks), with a resting period of a minimum of 2 weeks between sets. In total, the participants will undergo 30 sessions of Active or Sham tDCS (Figure 1).

This study will be conducted and reported according to the Consolidated Standards of Reporting Trials (CONSORT).

### 2.2 Recruitment and study setting

The study is expected to take place in the following hospitals: the Catholic University of Korea, Yeouido St. Mary’s Hospital (Seoul, Republic of Korea); the Catholic University of Korea, Seoul St. Mary’s Hospital (Seoul, Republic of Korea); and the Catholic University of Korea, St. Vincent’s Hospital (Suwon, Republic of Korea). Each hospital will screen and recruit volunteers who meet all the inclusion criteria and none of the exclusion criteria below (See Table 1).

**Table 1:** Schedule of enrollment, interventions, and assessments.

Schedule	Screening	Baseline	tDCS intervention: Set 1		Resting period	tDCS intervention: Set 2	
			3 weeks: 15 sessions	First Post-test		3 weeks: 15 sessions	Second Post-test
Visit	V1	V2	V2–V16	V16	Minimum 2 weeks ±7 days	V17–V31	V31
ENROLLMENT							
Informed consent	V						
Assigning Screening Numbers	V						
Demographic Survey	V						
Medical/surgical history	V						
Evaluation of inclusion/exclusion criteria	V	V					
Vital signs	V		V			V	
Anthropometry and physical examination	V	V	V			V	
Pregnancy test	V						
Laboratory tests	V						V
Prior/Concomitant Medications	V		V			V	
MRI scan	V						V
Randomization		V					
INTERVENTION							
Medical device preparation		V					
tDCS application			V			V	
ASSESSMENTS							
ADAS-Cog	V			V			V
K-MMSE	V			V			V
MoCA-K	V			V			V
HAM-D	V			V			V
S-IADL	V			V			V
Cortical Electric Field Assessment		V					
Adverse events scan			V			V	

tDCS, transcranial direct current stimulation; V, visit; MRI, magnetic resonance imaging; ADAs-Cog, Alzheimer’s disease assessment scale-cognitive subscale; K-MMSE, Korean version of Mini-Mental State Examination; MoCA-K, Korean version of Montreal Cognitive Assessment; HAM-D, Hamilton Depression Rating Scale; S-IADL, Seoul Instrumental Activities of Daily Living.

### 2.3 Participants

#### 2.3.1 Ethics approval

This protocol was approved by the Korean Ministry of Food and Drug Safety (MFDS) and will be conducted in accordance with the ethical standards of the 1964 Declaration of Helsinki. Additionally, the protocol was approved under the number XC23DSDS0053 by the Integrated Ethics Review Boards of the following universities: Catholic University of Korea, Yeouido St. Mary’s Hospital, Catholic University of Korea, Seoul St. Mary’s Hospital and Catholic University of Korea, St. Vincent’s Hospital.

Participants will sign an informed consent form after receiving a full description of the study’s objectives, benefits, and any potential discomfort they might experience during the intervention.

#### 2.3.2 Inclusion criteria

The inclusion criteria will be as follows: (1) individuals aged 60–85 years, both male and female; (2) individuals who meet the criteria based on the diagnostic guidelines provided by the National Institute on Aging and Alzheimer’s Association (NIA-AA) in 2011. Participants will have to meet all core clinical criteria for MCI due to AD and intermediate or high criteria for MCI based on integrated biomarkers. Criteria based on integrated biomarkers, intermediate: (a) Beta-amyloid biomarker positive, neurodegeneration biomarkers no detected, (b) Beta-amyloid biomarker no detected, neurodegeneration biomarker positive; and high: positive Beta-amyloid biomarker and positive neurodegeneration biomarker; (3) individuals with a score of 23 or higher in the Korean version of MMSE (K-MMSE); (4) individuals who meet one of the following criteria: clinical dementia rating global score of 0.5 or lower or Global deterioration scale score of 3 or lower; (5) If an individual is taking acetylcholinesterase inhibitors (ACEI) and N-methyl-D-aspartate (NMDA) receptor inhibitors, only those that have been on the same dosage and usage for at least 3 months from the screening date can be include in the study; (6) individuals taking medications for cognitive function other than ACEI and NMDA receptor inhibitors, as well as medications for chronic conditions such as depression, hypertension, diabetes, hyperlipidemia, and thyroid disorders, the dosage and usage of these medications must remain consistent for at least 1 month from the screening date; (7) individuals with the ability to read and comprehend the informed consent form and participant information sheet, and have sufficient language skills to respond to questionnaires; (8) individuals who voluntarily decide to participate in the clinical trial and provide written consent by signing the informed consent form; (9) individuals available to participate during the entire period of the trial.

#### 2.3.3 Exclusion criteria

The exclusion criteria will be as follows: (1) individuals diagnosed with dementia (major neurocognitive disorder) according to the criteria of DSM-5 (Diagnostic and Statistical Manual, fifth edition) or ICD-10 (The International Statistical Classification of Diseases and Related Health Problems); (2) individuals with CI due to the following conditions: Parkinson’s disease, Huntington’s disease, subdural hematoma, normal-pressure hydrocephalus, central nervous system infections (such as human immunodeficiency virus (HIV), syphilis), thyroid disorders, or deficiencies in vitamin B12 or folate; (3) individuals with a history of Axis I psychiatric disorders, including intellectual disability, schizophrenia, alcohol addiction, or bipolar disorder; (4) individuals with a history of seizures within 5 years from the screening date; (5) individuals who meet the following criteria based on computed tomography (CT) or MRI conducted within 1 year from the screening date: acute or subacute hemorrhage, previous large hemorrhage (defined as a diameter exceeding 1 cm on T2 sequence) or previous subdural hemorrhage without documentation indicating that it was not caused by underlying structural or vascular abnormalities, four or more microbleeds (defined as a diameter of 1 cm or less on T2 sequence), any single cortical–subcortical infarct larger than 1.5 cm (or 2 cm on diffusion-weighted imaging for contrast-enhanced MR), surface iron deposition, history of non-stenotic white matter disease defined as less than grade 3 on the age-related white matter change scale (ARWC), any finding that, in the opinion of the investigator, could potentially be a cause of dementia, pose a risk to the participant, or interfere with satisfactory MRI evaluation for safety monitoring purposes*; (6) individuals with brain damage due to trauma, ischemia, hypoxia, or other causes; (7) individuals who have been hospitalized for psychiatric or emotional disorders within 5 years from the screening date; (8) individuals with history of substance abuse within 5 years from the screening date; (9) individuals who have received treatment for alcohol addiction within 5 years from the screening date; (10) individuals whose visual impairment prevents them from reading ordinary writing even with glasses; (11) individuals whose visual hearing impairment makes them difficult to understand conversations even with hearing aids; (12) individuals with difficulty to breath while sitting still; (13) individuals with history of suicide attempts within 6 months from the screening date; (14) individuals with scalp deformities, inflammatory reactions, or other dermatological issues that would hinder EEG and tDCS electrode placement, as determined by a dermatologist; (15) individuals who meet to prohibitions on the use of tDCS medical devices, such as having metal plates inserted in the head, those who have participated in another clinical trial within 30 days from the screening date, those with a history of participation in a different clinical trial for MCI due to AD or prodromal AD within 1 year from the screening date; women of childbearing potential who do not consent to using medically acceptable methods of contraception during the duration of this clinical trial, pregnant or breastfeeding women, and any other clinically significant findings that, in the medical judgment of the principal investigator or responsible personnel, would make the individual inappropriate for this trial.

*In cases without CT or MRI results within 1 year of the screening date, imaging will be conducted to confirm the presence of brain disorders.

#### 2.3.4 Randomization

The participants will be randomized to the Optimized-tDCS and Sham-tDCS groups using a 1:1 ratio following the order of registration by a statistician who will not be involved in the application of the clinical trial. A stratified blocked randomization method with prespecified block sizes will be used. The stratification factor will be the hospital from which participants will be recruited. Random allocation numbers will be sealed in an opaque envelope and delivered to the medical device investigator who will administer the tDCS device in the hospital and will not be involved during the outcome measurement periods or other periods of the research.

### 2.4 Intervention

#### 2.4.1 Transcranial direct current stimulation

The tDCS device that will be used during the intervention period is a portable, battery-driven device called NEUROPHET innk (Neurophet, Seoul, Republic of Korea). The device can deliver a weak direct current between 1 and 2 mA for a programmed period through sponge-coated electrodes. In this study, participants will receive a stimulus of 2 mA for 30 min through two paired (one anode, one cathode) disk electrodes *R* = 1.5 cm.

The device is programmed to follow a ramping protocol: ramping up the current in 30 s to reduce discomfort at the beginning of the intervention and ramping down in 30 s at the end. Additionally, the device can continuously monitor the impedance values on the patient’s skin in real time. The stimulation is automatically terminated, and the medical device investigator verifies the patient’s condition and skin if an impedance of more than 13 Kohm is detected.

#### 2.4.2 Individualized 3D brain modeling

NEUROPHET tES LAB software (version 3.0; Neurophet, Seoul, Republic of Korea) will be used for brain modeling, calculate the tDCS-induced E-field and determine each participant’s optimal electrode location (according to individual brain structural characteristics) to stimulate a specific target area. In a recent simulation study, the optimized tDCS electrode position yielded a significant 55.28% increase in the E-field over the left dorsolateral prefrontal cortex (DLPFC) compared with the conventional 10–20 EEG-based system location (26). This emphasizes the potential of simulation software to enhance stimulation by optimizing the electrode locations in individual brain structures.

For the creation of the brain model and later analysis of the tDCS-induced E-field, all participants will undergo a T1-weighted MR image at baseline. The software will analyze and segment T1-weighted MR images and reconstruct a 3D model of the participant’s brain, including the following structures: skin, skull, cerebral gray and white matter, cerebellum gray and white matter, CSF, and ventricles. The electrical conductivity of the tissues is pre-programmed as follows: skin, 0.465 S/m; skull, 0.010 S/m; cerebral and cerebellar gray matter, 0.276 S/m; cerebral and cerebellar white matter, 0.126 S/m; and CSF and ventricle 1.65 S/m (27). Following the creation of the brain model, an investigator will assign landmarks (nasion, inion, and both preauricular points) to the model to assign the tDCS electrodes location.

#### 2.4.3 Optimized-tDCS group

For the optimized-tDCS group, guided by the 10–20 EEG-based system, the structure that represents the DLPFC will be selected on the brain model of each participant, as the DLPFC (F3) area is known for its direct relationship with MCI (28). Subsequently, a 3D model representation of an anode electrode will be located above the selected target, while a 3D model representation of the cathode electrode will be located over the contralateral supraorbital zone (Fp2), both electrodes will be disk type, *R*: 1.5 cm. Next, the tDCS intensity (2 mA) to be used in the study will be input into the software.

Based on these initial locations, tDCS parameters, and the conductivity of the brain tissues, the software analyzes the optimal electrode position for each participant. The F3 and Fp2 positions are used as references, as the software will “move” the electrodes around the target area to determine the location (according to the participant’s brain characteristics) that generates the maximum E-field-induced by tDCS. Finally, the software will provide the necessary guides to find the optimized electrode position on the participant’s head.

**Figure 2:**
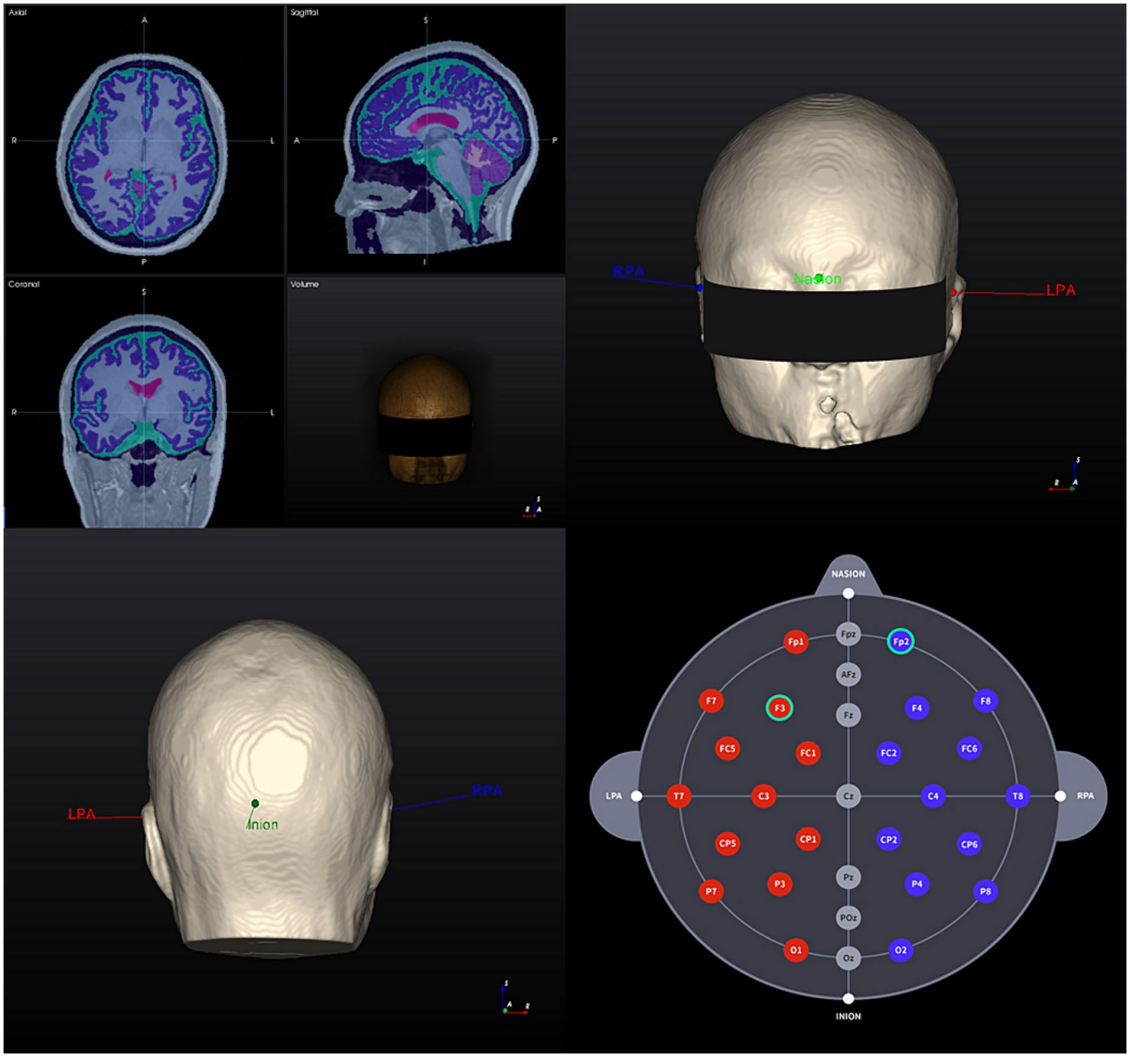
3D Brain model creation and landmarks 1. T1-weighted MR image of a participant (head model example) and segmentation results based on tissue electrical conductivity; 2. 3D brain model anterior view: landmark locations; 3. 3D brain model posterior view: landmark locations; 4. Individualized selection of tDCS electrode positions (F3 – Fp2). RPA, right pre-articular; LPA, left pre-articular.

**Figure 3:**
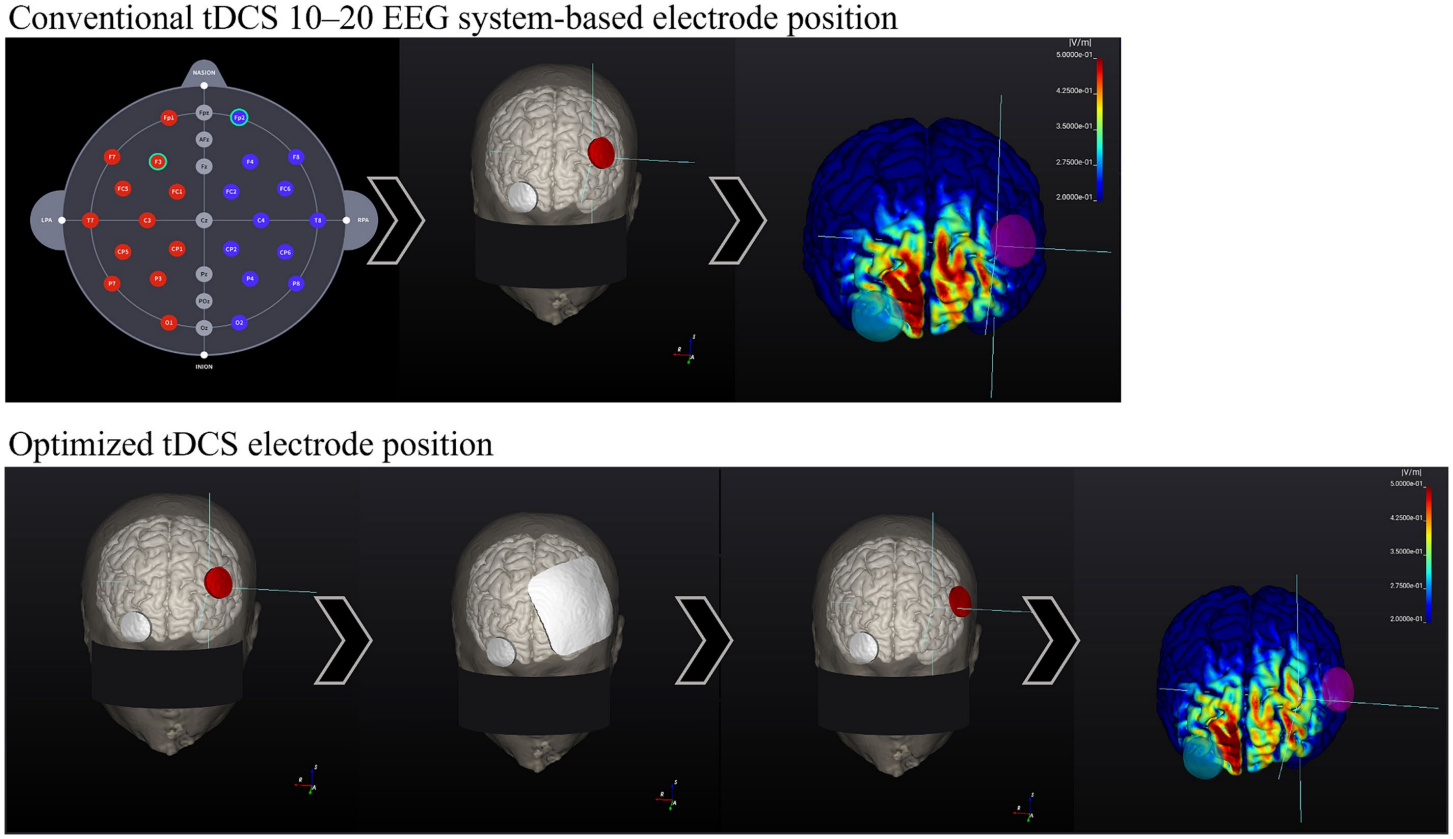
Comparison between 10–20 EEG system-based electrode location E-field and Optimized electrode location E-field. Head model example. Conventional tDCS 10–20 EEG system-based electrode position: (1.1) 10–20 EEG system-based electrode map. (2.1) Common electrode locations: F3 (anode, red) and Fp2 (cathode, gray). (3.1) Calculation of the E-field generated according to stimulation parameters. The E-field magnitude in the target zone (F3) was: 0.20 V/m. Optimized Active tDCS electrode position: (1.2) The target area is input into the program (F3), and the electrodes were initially located over the same locations as in conventional tDCS. (2.2) Based on the stimulation parameters, the program estimates the best electrode position to create the highest E-field magnitude on the target area. (3.2) After the calculations, the program shows the new personalized electrode position. (4.2) E-field magnitude generated according to the stimulation parameters and the optimized electrode position, E-field magnitude at the target zone (F3): 0.40 V/m.

**Figure 4:**
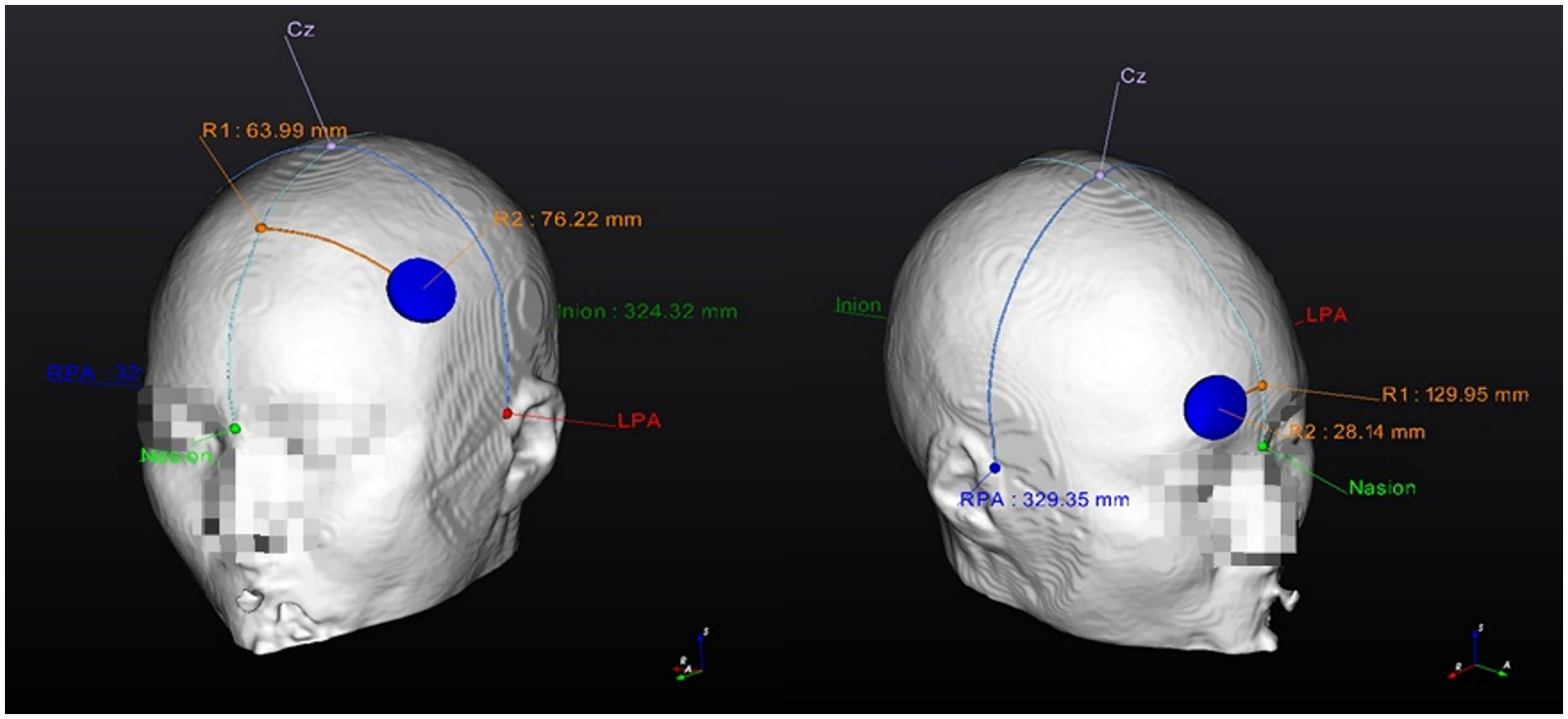
Personalized Optimized electrode position finding guide (example). 1. Anode location (F3); 2. Cathode location (Fp2). RPA, right pre-articular; LPA, left pre-articular.

Figures 2, 3 show visual representations of the NEUROPHET tES LAB program and the process to determine the optimized electrode locations. Figure 4 shows how the program offers the necessary guides to find the personalized electrode position on the participant’s head.

#### 2.4.4 Sham-tDCS group

In the case of the Sham-tDCS group, as the optimized-tDCS group, the software will provide each participant’s optimized tDCS electrodes position and the guides for the localization of the positions on the participant’s head. No stimulation will be performed. The device is going to ramp-up in 30 s and automatically ramp-down in another 30 s at the beginning and end of the stimulation to provide the initial sensation of tDCS.

### 2.5 Blinding

The participants and investigators in charge of performing the outcome measurements will remain unaware of the group allocation until the end of the study, and the data has been fully analyzed.

Owing to the nature of the study and individualized tDCS electrode localization, it is necessary that one investigator per hospital, in this case, the investigational device manager, remains unblinded during the investigation. The investigational device manager will be in charge of applying tDCS during the clinical trial; however, it will not be present during any outcome measurement period or at any other stage of the trial.

### 2.6 Outcome measurements

Outcome measurements will be obtained at three points: baseline (on the screening day), First Post-test (after the last session of the first set), and Second Post-test (after the last session of the second set) by investigators blinded to the participants’ allocation.

#### 2.6.1 Primary outcomes

##### 2.6.1.1 Alzheimer’s disease assessment scale-cognitive subscale

ADAS-Cog is considered the gold standard tool for assessing the effectiveness of anti-dementia treatment. It is frequently used in many observational and experimental studies, particularly in studies with patients ranging from those with normal cognition to MCI looking for treatments, to slow or stop the progression of the disease before it develops into severe neuropathology and dysfunction (29). The scale includes 11 tasks that include both participant-completed tests and observer-based assessments. Together, these tasks assess the cognitive domains of memory, language, and praxis. The specific tasks include word recall, naming objects and fingers, commands, constructional praxis, ideational praxis, orientation, word Recognition, and language. The total ADAS-Cog score ranges from 0 to 70, with higher scores indicating greater impairment (29). In this study, a validated Korean version of the test will be used (30).

##### 2.6.1.2 Korean version of mini-mental state examination

The K-MMSE is a Korean-validated version of the MMSE, which is a measurement tool designed for the quantitative estimation of CI severity and documentation of cognitive changes. It can be administered in 5–10 min, with questions grouped into seven categories, each representing a different cognitive domain or function: orientation to time, orientation to place, registration of three words, attention and calculation, recall of three words, language and visual construction. Scores range from 0 to 30, with lower scores indicating greater impairment (31, 32).

#### 2.6.2 Secondary outcomes

##### 2.6.2.1 Korean version of Montreal cognitive assessment

The MoCA-K is a Korean-validated version of the MoCA that is employed to assess and identify MCI. It can be administered in approximately 10 min. The MoCA-K assesses 11 domains, including attention and concentration, executive functions, memory, language, visuoconstructional skills, conceptual thinking, calculation, and orientation. The highest achievable score is 30 points, with lower scores indicating a greater CI (33, 34).

##### 2.6.2.2 Hamilton depression rating scale

HAM-D is a commonly used scale for assessing depression. Seventeen items are used to assess the severity of depressive symptoms: depressed mood, feelings of guilt, suicide, early insomnia, middle insomnia, late insomnia, work and activities, psychomotor retardation, psychomotor agitation, anxiety (psychic), anxiety (somatic), somatic gastrointestinal, somatic general, genital symptoms, hypochondriasis, weight loss, and insight. The overall score ranges from 0 to 52 points, with higher scores indicating more severe depression (35, 36).

##### 2.6.2.3 Seoul-instrumental activities of daily living

The S-IADL scale is designed to assess an individual’s capacity to perform instrumental and social activities of daily living. These include the ability to prepare balanced meals, remember appointments, keep financial records, and remember to take medications. It comprises 15 items with possible scores ranging from 0 to 45. Lower scores indicated better functioning (37).

##### 2.6.2.4 Imagining analysis

MR examinations for efficacy evaluation include 3D T1 MRI, resting-state functional MRI, and diffusion weighted image (DWI). The examinations will be performed at baseline and 8 weeks after tDCS application, and could be performed at screening instead of baseline or if results are available within 1 year of the consent date. 3D T1 MR scans will be acquired within a voxel size of 1 × 1 × 1 mm^3^, fMRI will be acquired at a resting state to analyze changes in neuronal efficiency, and diffusion tensor imaging (DTI) will be used to assess neural network connectivity.

### 2.7 Sample size

A prospective sample calculation was not performed because the current study protocol is for assessing feasibility, safety, and trends in effectiveness, which will be utilized to generate data for future full-scale randomized controlled trials. However, as sample sizes of a minimum of 24 participants (12 per group) were recommended, we decided to recruit a minimum of 32 participants based on the predicted recruitment rates within the study timeframe (38, 39). Considering a 20% dropout rate, the projected number of participants to be recruited will be 40.

### 2.8 Statistical analyses

Descriptive statistics (average, standard deviation, median, minimum, and maximum) will be represented for each group for the changes found from baseline to the first and second post-tests. The Least Squares Mean (LSM) with a 95% confidence interval will be used to determine the trend of changes within time per group. The Shapiro–Wilk test will be used to determine data normality. If normality is met, the paired *t*-test will be used for within-group analysis at each time point; if normality is not met the Wil-coxon sign rank test will be performed. Following this, an independent *t*-test will be used for between-group analysis; if normality is not met, a Wilcoxon sign rank test would be performed. Additionally, an analysis of covariance (ANCOVA) with baseline as a covariate will be performed for a group × time analysis.

### 2.9 Safety assessment protocol

The safety assessment protocol includes the registration of all the adverse events that may arise during the clinical trial. Additionally, vital signs, physical examinations, and monitoring of concomitant medications will be performed to assess the safety.

#### 2.9.1 Treatment emergent adverse events assess

Treatment-emergent adverse events (TEAE) will be assessed from the initial application of the trial until its end. In this study, TEAE means an adverse event that occurred after the application of an investigational device, that is after tDCS application. TEAE events related to tDCS will be identified during and up to 30 min after each tDCS application.

All TEAE will be standardized using System Organ Class (SOC) and Preferred Term (PT) in the most recent version of the Medical Dictionary for Regulatory Activities (MedDRA). If the same TEAE occurs multiple times in one person, it will be classified as a single case; however, if the severity of the same TEAE varies, only the maximum severity TEAE will be recorded. If the causality of the same adverse event differs for the same person, only the adverse events that appear to be caused by tDCS will be recorded. The number of subjects with TEAEs, number of events and percentage per events will be registered and organized into adverse device events (ADEs), serious adverse events (SAEs), and serious adverse device events (SADEs).

Additionally, the differences between groups will be tested using the Pearson’s chi-square test (or Fisher’s exact test if the expected frequency is less than five cells and exceeds 20%). After standardizing the latest version of the MedDRA code, SOC, and PT, TEAE’s occurrences will be reported for each group.

#### 2.9.2 Vital signs examination

Vital signs will be measured at baseline and at the first and second post-tests. The following parameters will be evaluated: systolic/diastolic blood pressure (measured after 5 min of sitting), pulse, and body temperature.

#### 2.9.3 Concomitant medications

Patients undergoing conventional cognitive rehabilitation therapy will be able to participate in the trial without interrupting their therapy. Participants may be allowed to receive concomitant surgery, medication, or other medical devices only if the investigator believes that it will not affect the interpretation of the findings from the study. The investigator will assess the method and frequency of cognitive rehabilitation therapy as well as the type and dosage of all concomitant medications during the trial period at each visit and record them in a case record form (CRF) to monitor and identify adverse events. If the number of medications changes before and after participation in this study, a principal investigator will check for changes in the participant’s underlying medical condition. If the principal investigator determines that the change is a meaningful clinical change that could affect the study results, the data will be excluded from the main analysis group.

The number of participants, percentage, and number of concomitant medications will be reported and intergroup differences will be compared using the chi-square test (or Fisher’s exact test if the expected frequency is less than five cells and exceeds 20%). We will also analyze the correlation between the number and type of concomitant treatments and tDCS outcomes. Additionally, after standardizing the most recent version of the Anatomical Therapeutic Chemical Classification (ATC) code, the number of participants, percentages, and incidences of concomitant medication usage will be reported by group.

## 3 Trial status

Participant recruitment began in August 2023 and is expected to be complete (including follow-up testing) by October 2024.

## 4 Discussion

The present randomized double-blind controlled trial aims to prove the feasibility and safety of optimized tDCS in the treatment of patients with MCI-induced by AD for cognitive function improvement, throughout the use of 3D brain models based on personalized MRI scans to provide optimized tDCS electrode locations per participant.

The advancement of MCI induced by AD leads to great economic burden and limitations during daily life activities of the patients who suffer from it, who are commonly older adults who will require treatment for the rest of their lives (40). Hence, it is necessary to search for a treatment that aids in improving cognitive function while being safe for long-term use. Previous research has shown that tDCS can be safely used for 20 days and has significant effects on cognitive function (11, 12). Although there is a study that applied tDCS for 6 months in patients with MCI, more research is necessary because of the small sample size (41). Hence, the aim of the present study to apply tDCS to a relatively large sample of over 30 sessions, which is highly relevant for the future use of tDCS in this population.

tDCS has shown mixed results regarding its effectiveness in improving cognitive function. A recent study reported that tDCS over the DLPFC at 2 mA for 20 min in older adults with MCI due to AD effectively improved general cognition and immediate memory, as assessed using the MMSE (11). A systematic review showed that anodal tDCS may be an effective adjunctive therapy for patients with MCI (2). However, another systematic review reported inconsistencies regarding its effectiveness (8). Furthermore, a study applied tDCS twice a day for five consecutive days and found no significant changes following the results of ADAS-Cog and MMSE tests. The authors of a previous study hypothesized that the presence of patients with MCI and major cognitive disorders in the sample might have influenced the study results.

The results of past research and the inconsistencies reported in the systematic review can be attributed, in part, to inter-individual factors. The progression of MCI to a major cognitive disorder produces changes in certain brain structures (unique to each individual), such as generalized cortical atrophy characterized by reduced gyri volume, increased sulci width, and enlarged ventricles (especially the lateral ventricle) (15). Furthermore, an increase in the size of the ventricles appears to be a primary sign or secondary effect associated with atrophy of the periventricular parenchyma (15). Brain tissue atrophy caused by age-related changes and conditions, such as MCI, can disrupt current tDCS pathways. To investigate this further, one study used three structural MRI scans to develop 3D brain models representing a young adult, an older adult, and an MCI patient and subsequently applied the same tDCS protocol to each of the models. They found that the maximum current density in the cortical tissue decreased according to the degree of GM atrophy (42).

Therefore, it is clear why individual brain characteristics must be considered when designing tDCS interventions. MRI-based 3D brain models for optimized tDCS electrode location may be a solution for these inter-individual factors; hence, the application of this study will be of significant importance for the future use of tDCS in this population and the assessment of its effects.

According to previous research, the use of a fixed, classic location for the tDCS electrodes does not consider individual differences in anatomy, limiting the amount of current reaching the brain and causing variability in the E-field at the cortical target site, influencing the tDCS effects (43). One study assessed the correlation between E-field strength and the improvement of working memory (WM) in older adults after 10 sessions of 2 mA tDCS over the classic DLPFC locations, finding that participants with an E-field higher than average within the study sample had an increased WM improvement after the intervention (44). Although it is a different population, these results prove that ensuring the most adequate E-field per person could positively impact the effectiveness of tDCS applied to the DLPFC.

In addition, recent research has found that age and biological sex influence the current density at a target point. A study using 3D modeling based on approximately 700 individuals’ MRIs determined that older female adults received a higher simulated current at the DLPFC target area than their counterparts using the same tDCS protocol. They concluded that individual modeling is required to account for the variability in cortical morphometry and to adjust the stimulation parameters of tDCS to achieve the intended stimulation benefits (45).

Moreover, the effect of tDCS is influenced by electrode characteristics (46). Generally, most tDCS studies have used electrode sizes of 25 cm^2^ to 35 cm^2^ with currents between 1 and 3 mA and a duration of 20–30 min. Large electrodes stimulate large regions of the brain and computer simulation studies have shown no focal current density distribution. In addition, these electrodes contribute to the edge effect, which is the current concentration at the edges of the electrodes at the corners of the rectangular and square electrodes. Using smaller and circular electrodes may help decrease edge effects and improve the focus of the current in the target area (46). Therefore, 1.5 cm disk electrodes will be employed in this study.

The use of tDCS for 20 days in patients with MCI has been proven to be safe (5). Moreover, one study safely applied tDCS for six months in patients with AD at an intensity of 2 mA for 30 min (41). However, these previous studies used wider electrodes (rectangular 7 × 5 cm and round 6 cm diameter, respectively) than those used in the present study. In a study with healthy volunteers, one session of 1 mA for 12 min was applied using electrodes (3.14 cm^2^) to the sensorimotor area without reporting adverse effects; to the best of our knowledge, this is the only study that used two electrodes of a similar small size to ours (47). Our study will use small disk electrodes for a relatively long period (30 sessions in total); therefore, as the safety of this electrode size in an MCI population has not yet been reported, the decision to divide the 30 sessions into two blocks of 15 sessions with a resting period in between was made.

According to a previous study, five consecutive sessions of tDCS in patients with MCI at 2 mA for 20 min over F3 significantly improved delayed recall, with results persisting after one month of follow-up (48). Another study applied tDCS in patients with AD for 25 min daily (10 sessions) at 2 mA to determine its effectiveness in treating cognitive decline, and found that the MMSE scores showed significant changes that were maintained at 1 and 2 months after the intervention (49). According to previous research, the effects of tDCS can be maintained for at least one month after five sessions, which shows that a resting period of two weeks is sufficient without diminishing the effectiveness of tDCS.

To the best of our knowledge, personalized bilateral optimization of tDCS in patients with AD-induced MCI has not been reported. Nevertheless, a similar study applied six sessions of personalized optimized anodal high-definition tDCS at 2 mA for 20 min to adults with AD, which resulted in significant improvement of the MMSE scores in comparison with the sham group (16). However, we believe that the validity of bilateral tDCS with two electrodes still needs to be validated and this trial aims to validate the safety and efficacy of personalized tDCS as a new tDCS protocol.

The trial presents various limitations. First, the absence of a group using conventional tDCS electrode locations guided by the 10–20 EEG-based system for comparison between optimized and conventional tDCS electrode locations. After concluding this trial, we will consider including a conventional tDCS group in future studies. Second, no previous studies have directly supported the safety of the electrode type chosen in this study. Nevertheless, this study will be able to validate its efficacy and safety relative to sham tDCS. Third, the absence of a follow-up evaluation to determine if the effectiveness of the treatment will be maintained in time. A follow-up evaluation will be considered in future fully powered trials.

For this present study, we hypothesize that the participants will tolerate the optimized tDCS intervention without any significant adverse effects, and that, compared to the sham tDCS group, the optimized personalized tDCS application will affect the treatment of patients with MCI-induced AD for cognitive function.

## 5 Ethics and dissemination

This protocol was approved by the MFDS and will be conducted according to the ethical standards of the 1964 Declaration of Helsinki. The protocol was approved by the Ethics Review Boards of the three universities mentioned in the Methods and Analysis section. In addition, the participants will sign an informed consent form to participate in the intervention.

## Ethics statement

This protocol was approved by the Korean Ministry of Food and Drug Safety (MFDS) and will be conducted in accordance with the ethical standards of the 1964 Declaration of Helsinki. Additionally, the protocol was approved under the number XC23DSDS0053 by the Integrated Ethics Review Boards of the following universities: Catholic University of Korea, Yeouido St. Mary’s Hospital, Catholic University of Korea, Seoul St. Mary’s Hospital and Catholic University of Korea, St. Vincent’s Hospital. Participants will sign an informed consent form after receiving a full description of the study’s objectives, benefits, and any potential discomfort they might experience during the intervention.

## Author contributions

TK: Conceptualization, Methodology, Writing – original draft, Writing – review & editing. DWK: Conceptualization, Funding acquisition, Methodology, Writing – review & editing. JCSF: Writing – original draft, Writing – review & editing. HJ: Conceptualization, Methodology, Writing – original draft. YU: Conceptualization, Writing – review & editing. SK: Conceptualization, Writing – review & editing. S-MW: Conceptualization, Writing – review & editing. DK: Project administration, Supervision, Writing – review & editing. HL: Funding acquisition, Project administration, Supervision, Writing – review & editing.
